# Semiconductor Metal Oxides as Chemoresistive Sensors for Detecting Volatile Organic Compounds

**DOI:** 10.3390/s19020233

**Published:** 2019-01-09

**Authors:** Tingting Lin, Xin Lv, Zhineng Hu, Aoshu Xu, Caihui Feng

**Affiliations:** 1 College of Instrumentation and Electrical Engineering, Jilin University, Changchun 130061, China; xinlv15@mails.jlu.edu.cn (X.L.); huzn17@mails.jlu.edu.cn (Z.H.); xuks15@mails.jlu.edu.cn (A.X.); 2 Key Laboratory of Geophysics Exploration Equipment, Ministry of Education of China, Changchun 130061, China

**Keywords:** volatile organic compounds, semiconductor, metal oxide, gas sensor, sensitivity

## Abstract

Volatile organic compounds (VOCs), which originate from painting, oil refining and vehicle exhaust emissions, are hazardous gases that have significant effects on air quality and human health. The detection of VOCs is of special importance to environmental safety. Among the various detection methods, chemoresistive semiconductor metal oxide gas sensors are considered to be the most promising technique due to their easy production, low cost and good portability. Sensitivity is an important parameter of gas sensors and is greatly affected by the microstructure, defects, catalyst, heterojunction and humidity. By adjusting the aforementioned factors, the sensitivity of gas sensors can be improved further. In this review, attention will be focused on how to improve the sensitivity of chemoresistive gas sensors towards certain common VOCs with respect to the five factors mentioned above.

## 1. Introduction

Volatile organic compounds (VOCs) are common in the environment and are encountered in many activities, such as painting a house, cooking, driving a car, oil refining and cutting the grass, etc. [1,2,3]. In other words, anthropogenic sources increase the release of VOCs beside natural sources. The increased emissions of VOCs have an adverse influence on the ambient air [4,5,6,7,8]. Moreover, certain volatile organic compounds are identified as hazardous gases and can cause severe diseases such as allergy, colon cancer, rectal cancer, and lung cancer [9]. Therefore, environmental monitoring is essential for evaluating the air quality [10,11,12,13,14]. 

Currently, effective analytical techniques, including gas chromatography and spectrometry, have been used to monitor VOCs [15,16]. Although these analytical techniques are sufficiently accurate, they are expensive, time-consuming and lack portability. Therefore, many researchers have attempted to design effective, cheap, fast response and small gas sensors for the quantification of VOCs. Based on the different sensing mechanisms, gas sensors are divided into several main types, including resistive, catalytic, optical and electrochemical [17]. Among them, resistive gas sensors based on semiconductor metal oxides have been considered attractive candidates for detecting hazardous VOCs due to the fast response and recovery time, ease of use, low-cost, high sensitivity and good portability [18,19,20,21,22,23,24]. 

Over the past decades, researchers have been working to improve the gas-sensing performance of semiconductor metal oxide gas sensors [25,26,27,28,29,30,31,32,33]. Sensitivity is one of the major parameters of semiconductor metal oxide gas sensors and is affected by many factors [34,35]. Although several reviews have been published on semiconductor metal oxide gas sensors, it is still necessary to summarize the factors affecting the sensitivity for further improvement of the gas-sensing performance. Herein, we intend to provide a review on chemoresistive semiconductor metal oxide gas sensors for detecting VOCs, including ethanol, acetone, formaldehyde, toluene and acetylene. First, we introduce five different VOC gases and briefly describe the gas-sensing mechanism of chemoresistive semiconductor metal oxide gas sensors. Then, we summarize the five factors, including the microstructure, defect, catalyst, heterojunction and humidity that affect gas sensitivity. Finally, we review the recent developments in chemoresistive semiconductor metal oxide gas sensors towards five different kinds of VOCs.

## 2. Volatile Organic Compounds in the Environment 

In general, organic compounds consist of at least one carbon and one hydrogen atom. On the basis of volatility, organic compounds are divided into three types: non-volatile organic compounds (NVOCs), semi-volatile organic compounds (SVOCs) and volatile organic compounds (VOCs) [33]. There are many definitions of VOCs according to different considerations. The U.S. Environmental Protection Agency (USEPA) defines VOCs as follows: “any carbon compounds excluding carbon monoxide, carbon dioxide, carbonic acid, metallic carbide, metallic carbonate and ammonium carbonate, which participate in atmospheric photochemical reactions” [29]. The World Health Organization (WHO) defines VOCs as: “organic compounds having the melting point below room temperature and the boiling point between 50–260 °C”. However, China describes VOCs as “any organic chemical compounds that are easy to volatilize at normal pressure and temperature” [1].

Table 1 shows the chemical formula, formula weight, IDLH (immediately dangerous to life or health) concentration and TLV (threshold limit value) of VOCs. The TLV refers to the maximum concentration at which repeated exposure to VOCs can be endured without any adverse health effects [36,37].

### 2.1. Ethanol

Ethanol is a volatile, colourless transparent liquid with a melting point of –114 °C, and a boiling point of 78.3 °C. Ethanol is widely used in the fields of the defence industry, food industry, industrial and agricultural production as well as medical and health services. Ethanol has a particular smell, and can be used to make acetic acid [38], beverages [39] and essences [40]. In addition, ethanol is the raw material for producing dyes, paints and cosmetics. In the medical field, ethanol is also used as a medical disinfectant. Upon exposure to ethanol vapor, it may cause dizziness, headache, fatigue and nausea [41]. More importantly, ethanol vapor and air may form an explosive mixture, so it is necessary to monitor the concentration of ethanol in industrial production and road transportation practices.

### 2.2. Acetone

Acetone is a representative type of ketone. It is a clear and colourless liquid with a pungent odour. As a reagent, acetone is widely used as a diluting agent, cleaning agent and extraction agent in laboratories. Acetone is also used as an effective solvent in a range of industrial products, such as paints and agglomerants. The compound, which is one of the most essential raw materials for organic synthesis, is used to produce epoxy resins, polymethyl methacrylate and pharmaceutics. Acetone can irritate the throat, nose and eyes [42,43]. Long-term exposure to acetone can result in pharyngitis, bronchitis and dermatitis. The quantitative detection of acetone is important for good health [44].

### 2.3. Formaldehyde

Formaldehyde has been widely used in many industries, such as the furniture, textile and printing industry [45]. It is a stimulating, colourless, pungent-smelling gas, easily soluble in water. Exposure to low levels of formaldehyde can lead to a variety of physical discomforts, such as dizziness, fatigue, chest distress, sore eyes, palpitation and nausea. Upon exposure to high levels of formaldehyde, the inhalation of gas can cause difficulty in breathing and lung oedema [46]. Due to the toxicity of formaldehyde, the Occupational Safety and Health Administration (OSHA) has established the TLV as a concentration of 0.75 ppm for 8 h. Indoor formaldehyde, which is emitted from antiseptics, glues, plastics, wood furniture and carpet cleaners, is closely related to people’s daily life [47]. For environmental and safety purposes, manufacturing gas sensors for formaldehyde detection is urgent. 

### 2.4. Toluene

Toluene is a clear, colourless volatile liquid with a particular aromatic odour, and is insoluble in water, but soluble in ethanol and acetone [48]. Toluene is one of the most widely used aromatic hydrocarbons and has many applications in the manufacture of adhesives, dyes, medicine, pesticides, rubber, fingernail polish and explosive materials [49]. In urban areas, toluene is emitted in processes related to automobile exhaust and gasoline. Moreover, toluene is an irritant to the skin and the mucous membrane. At higher concentrations, toluene can cause nervousness, restlessness and a rapid heartbeat. Toluene may also cause damage to the liver or kidneys. The OSHA has defined the permissible exposure limit for toluene as 100 ppm for 8 h [50]. Toluene is considered a kind of cancer biomarker. In fact, high levels of toluene can be detected in the exhaled breath of people with lung cancer compared to healthy non-smokers. Hence, the development of a high performance toluene sensor is not only of medical but also of social importance.

### 2.5. Acetylene

Acetylene is a reactive and unstable hydrocarbon gas [51]. It is a raw material of acetaldehyde, acetic acid, benzene, synthetic rubber, paints, dry-cleaning solvents and insecticide sprays [52]. In addition, the combustion of the compound is used for illumination, cutting metals and oxyacetylene welding [53]. However, acetylene can be explosive in the pressurized form and in the presence of electric sparks. Acetylene has a weak narcotic effect and prevents cell oxygenation. Upon exposure to high levels of acetylene, oxygen in the air is forced out, resulting in suffocation.

## 3. Gas-Sensing Mechanism of Semiconductor Metal Oxide Sensors towards Volatile Organic Compounds

To fabricate high performance sensors that are used for monitoring VOCs, it is necessary to understand the gas-sensing mechanism. In general, a gas sensor based on semiconductor metal oxides can be applied to detecting the target gas via a redox reaction between the gas molecules and the sensing material [54]. In the presence of air, oxygen gas is absorbed on the surface of the sensing material, resulting in adsorbed oxygen. Due to different working temperatures, adsorbed oxygen are different oxygen species including O_2_^−^, O^−^ and O^2−^ [55]. The adsorption kinematics are described as the follows:

(1) $$O_{2}\left(\mathrm{gas}\right)\to O_{2}\left(\mathrm{absorbed}\right)$$

(2) $$O_{2}\left(\mathrm{absorbed}\right)+e^{-}\to O_{2}{}^{-}$$

(3) $$O_{2}{}^{-}+e^{-}\to {2O}^{-}$$

(4) $$O^{-}+e^{-}\to O^{2-}$$

The formation of oxygen species results in the capture of electrons from the conduction band, leading to a change in conductivity of the semiconductor metal oxide [56]. The variation in conductivity results from a change in the charge carrier concentration. According to the different types of major carriers, semiconductor metal oxide is classified into n-type and p-type. The charge carriers of n-type semiconductor metal oxide are electrons, so the interaction between the semiconductor metal oxide and the ionic oxygen species causes a decrease in conductivity upon exposure to air. In contrast, the charge carriers of p-type semiconductor metal oxide are holes, resulting in an increase in conductivity [57].

**Figure 1:**
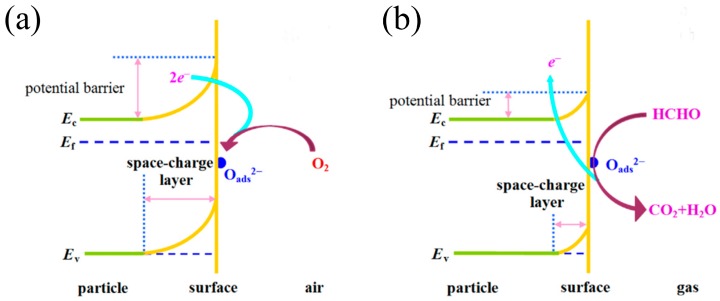
A schematic diagram of reaction mechanism of SnO_2_-based sensor to HCHO: (**a**) in air, (**b**) in VOC. Reprinted from [61] with permission.

The well-accepted gas-sensing mechanism is described as a change in conductivity of the sensor [58,59,60]. Herein, a brief introduction to the sensing mechanism of n-type semiconductor metal oxide is given based on the example of SnO_2_, as shown in Figure 1 [61]. In air, the oxygen molecules are adsorbed on the surface of semiconductor metal oxide, forming a potential barrier. The interaction between oxygen molecules and semiconductor metal oxide forms charged oxygen species by capturing electrons from the conduction band, leading to an electron-depleted region and band bending on the surface of the semiconductor metal oxide. The electron-depleted region is also referred to as the space-charge layer, as shown in Figure 1a. Its thickness is the length of the band bending region. The variation in band bending increases the resistance of the sensor. When the sensor is exposed to HCHO, HCHO will react with the adsorbed oxygen ions and release the trapped electrons back to the semiconductor metal oxide, resulting in an increase in the carrier concentration. As a consequence, the thickness of the space-charge layer is reduced, resulting in a decrease of the potential barrier and resistance, as shown in Figure 1b.

## 4. Factors Affecting the Sensitivity of Semiconductor Metal Oxide Gas Sensors

Sensitivity is a parameter reflecting the resistance variation in a certain concentration of target gas. For n-type semiconductor metal oxide sensors, the sensitivity is defined as R_g_/R_a_ for oxidizing gases and R_a_/R_g_ for reducing gases, where R_a_ and R_g_ are the resistances of the gas sensors in the presence of air and target gases, respectively. However, the sensitivity of p-type semiconductor metal oxide sensors has the opposite definition. The enhancement of sensitivity is of great importance in obtaining excellent gas sensors. Researchers have devoted great efforts to fabricate high performance gas sensors [62,63,64,65,66,67,68,69,70,71]. The sensitivity of semiconductor metal oxide gas sensors can be improved by adjusting the microstructure, defects, catalyst, heterojunction and humidity.

### 4.1. Effect of the Microstructure

The response of semiconductor metal oxide sensors is based on the interaction between the target gases and adsorbed oxygen species. The adsorption-diffusion-desorption process occurs on the surface of the semiconductor metal oxide sensor. Therefore, the gas-sensing performance is influenced by the morphology of the semiconductor metal oxide. The optimal morphology promotes the adsorption and desorption of the adsorbed oxygen species, which plays a crucial role in the enhancement of the gas-sensing performance. Thus, it is necessary to understand the parameters of the microstructure for further improvement of gas sensors [25]. The grain size, number of activated adsorption sites and gas diffusion ability are the structural parameters that dramatically affect the gas-sensing performance.

#### 4.1.1. Grain Size

**Figure 2:**
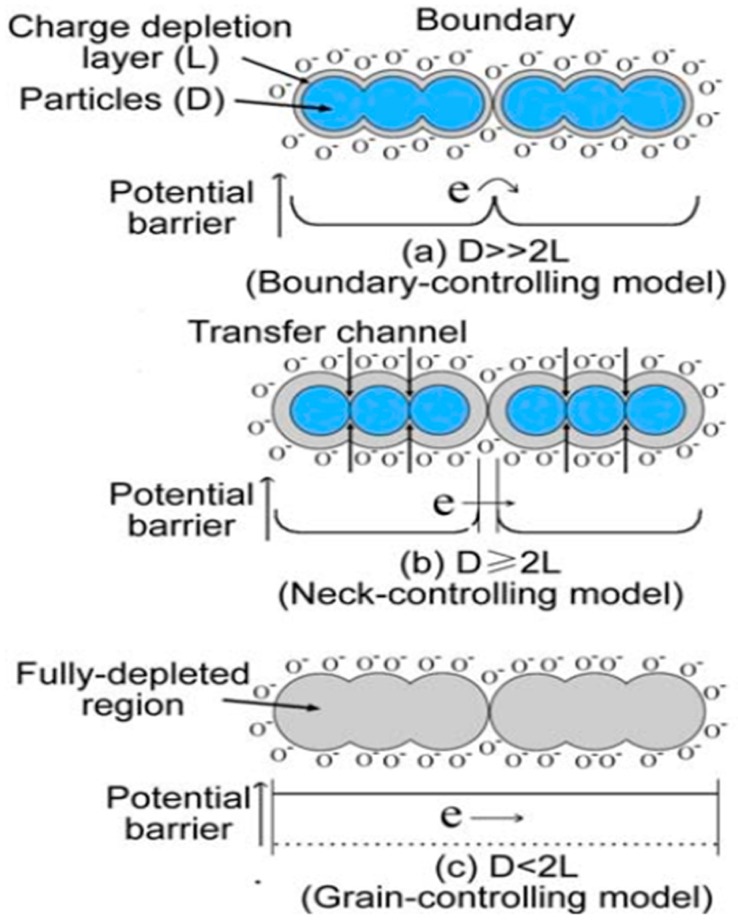
Schematic model of the effect of the crystallite size on the sensitivity of semiconductor metal oxide gas sensors: (**a**) D >> 2 L; (**b**) D ≥ 2 L; (**c**) D < 2 L. Reprinted from [28] with permission.

The reduction in grain size to the nanoscale is one of the most effective strategies for the enhancement of the gas-sensing properties. In other words, the sensitivity of a gas sensor is directly affected by the grain size [72]. To gain insight into the impact of the grain size on sensitivity, Xu et al. proposed a model to illustrate the remarkable grain size effect [73]. As shown in Figure 2, the sensing material was composed of partially sintered grains connected to each other by necks [28]. 

The interconnected grains formed larger aggregates connected to each other by the grain boundaries. Generally, there are three cases with respect to the relationships between the grain size (D) and the width of the space-charge layer (L), which are described in terms of the boundary control, neck control and grain control. For large enough grains (D >> 2 L), most grains were unaffected by the surface interactions with the gas phase. The predominant impact of gas on the conductivity of the sensor was provided by the grain boundary barriers, as shown in Figure 2a. Therefore, the grain boundary controlled the gas-sensing mechanism. The sensitivity of the sensing material was independent of the grain size. For grains with D ≥ 2 L, the space-charge layer around each neck formed a conduction channel, as depicted in Figure 2b. The conductivity could be decided collaboratively by the cross-sectional area of those channels and the grain boundary barriers, resulting in an enhanced sensitivity. Furthermore, the sensitivity of the sensing material became dependent in the grain size and increased with the reduction in the grain size. For grains with D < 2 L, the space-charge layer extended throughout the whole grains. As seen from Figure 2c, the grains were almost fully depleted of mobile charge carriers. The conduction channels between the grains were mislaid, leading to a dramatically decreased conductivity. Due to the lack of significant barriers for the interconnected grain charge transport, the energy bands were nearly flat throughout the grains. Clearly, the sensitivity of the sensing material was controlled by the grains. It was found that the highest sensitivity was achieved in the case of D < 2 L. Based on Xu’s model, Liang et al. have prepared highly monodisperse α-Fe_2_O_3_ nanoparticles with an average particle size of 3.1 nm, which exhibited an excellent acetone sensing performance due to the relation between the grain size (D) and the width of the space-charge layer (L) according to D < 2 L [74]. The almost fully depleted grains induced changes to the overall conductivity, leading to improvement of the sensitivity.

#### 4.1.2. Number of Activated Adsorption Sites

**Figure 3:**
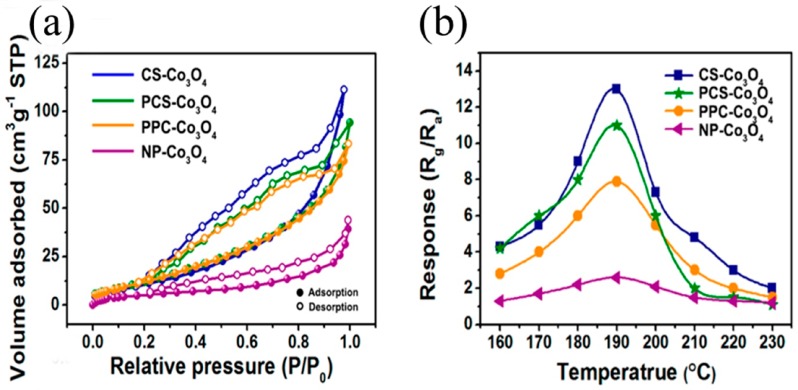
(**a**) N_2_ adsorption-desorption isotherms of Metal-Organic Frameworks-derived Co_3_O_4_ structures; **(b**) Responses of CS-, PCS-, PPC-, and NP-Co_3_O_4_-based sensors at different operating temperature toward 200 ppm acetone; Reprinted from [75] with permission.

It is well acknowledged that the sensing reaction of a gas sensor occurs at the active sites on the semiconductor metal oxide surface. Therefore, the number of activated adsorption sites has a great influence on the improvement of the gas-sensing performance. One good strategy to increase the number of active sites is the adoption of increased the specific surface area, which has a potentially strong impact on the adsorption ability of gas sensors. Accordingly, many researchers have strived to increase the specific surface area for obtaining enhancement of the sensitivity. For example, Zhang et al. synthesized Co_3_O_4_ products based on the core-shell (CS) nanostructure, porous core-shell (PCS) nanostructure, porous popcorn (PPC) nanostructure, and nanoparticle (NP) nanostructure to verify that the specific surface area affected the sensitivity of gas sensors [75]. As shown in Figure 3a, the specific surface areas of CS-, PCS-, PPC-, and NP-Co_3_O_4_ were 44.5, 42.8, 42.4 and 12.2 m^2^·g^−1^, respectively. The response values of CS-, PCS-, PPC-, and NP-Co_3_O_4_ towards 200 ppm acetone were 13, 11, 7.9, and 2.6, respectively (Figure 3b). Obviously, the larger specific surface endowed the Co_3_O_4_ nanostructures with more activated adsorption sites, thereby increasing the adsorption of gas molecules and improving the sensitivity of the gas sensor.

#### 4.1.3. Gas Diffusion

Except for the large specific surface area, enhancing the gas diffusion of the sensing materials is one strategy that can improve the gas-sensing performance. Generally, the sensing materials of gas sensors are classified into dense and porous nanostructures. In the case of dense nanostructures, gas diffusion can only occur on the surfaces of sensing materials because the gas molecules cannot penetrate into the sensing materials. In the case of porous nanostructures, the gas molecules can interact with the inner grains because the special structures are conducive to the penetration of gas molecules into the sensing materials. Sufficient diffusion can lead to a large change in the resistance of the gas sensor. The porous structure is divided into microporous, mesoporous and macroporous structure based on the pore size. The pore size of a microporous structure is smaller than 2 nm, whereas the pore size of a mesoporous structure ranges from 2–100 nm. The macroporous structure has a pore width larger than 100 nm. The porosity of semiconductor metal oxide has a profound effect on the gas diffusion depth, thereby affecting the sensitivity of the gas sensor. Sakai et al. proposed a theory of gas diffusion controlled sensitivity [76]. The diffusion of mesoporous nanostructures could be described by the Knudsen diffusion. The Knudsen diffusion coefficient (D_k_) is related to the pore width (r), temperature (T) and molecular weight (M) of gas molecule:

(5) $$D_{k}=\frac{4r}{3}\sqrt{\frac{2\mathrm{RT}}{\pi M}}$$

**Figure 4:**
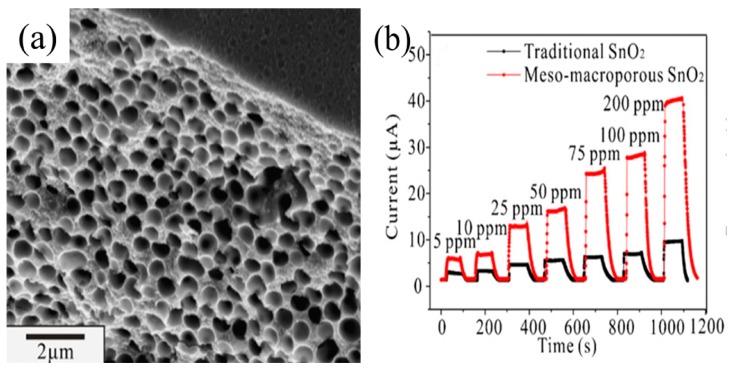
(**a**) SEM image of meso-macroporous SnO_2_; (**b**) Real-time responses of the sensors based on meso-macroporous SnO_2_ and traditional SnO_2_, respectively. Reprinted from [77] with permission.

The response of mesoporous nanostructures varied directly with the Knudsen diffusion coefficient (D_k_) and inversely with the thickness (L) of the sensing film. Notably, the gas diffusion increased with increasing pore radius, contributing to an increase in the sensor response. In addition to Knudsen diffusion, there was another type of gas diffusion as the pore size was larger than 100 nm. The alternative type of gas diffusion allowed gases to diffuse more quickly through molecular diffusion, which is analogous to reducing the thickness (L) of the sensing film, thereby enhancing the sensor response. This process was recently proven by an experimental study in which the sensor response was significantly improved by using a porous structure with meso- and macroporosity, as shown in Figure 4 [77].

### 4.2. Effect of the Defects

Defects have crucial impacts on the physical characteristics and electronic structures of semiconductor metal oxides, thereby playing an important role in the sensing reaction. Previous reports have shown that defects are beneficial to the improvement of sensitivity. Notably, manipulating the defect structures of semiconductor metal oxides can effectively enhance the gas-sensing performance. Li et al. established a relation between the defect structure and the sensor response. ZnO nanosheets were prepared by the precipitation method at different calcination temperatures (200, 400 and 600 °C), which were exploited to detect acetone. Li et al. found that the ZnO nanosheets, which had been calcined at 200 °C, showed high acetone sensitivity [78]. The superior performance might be attributed to the morphology, grain size, specific surface area and surface defect content. Notably, the shape and grain size of the ZnO nanosheets did not change below 400 °C. As a consequence, these influencing factors were ruled out simultaneously. Nevertheless, the specific surface area of ZnO nanosheets at different calcination temperatures was different, and the values could impact the performance. Therefore, the effect of the specific surface area was excluded by normalized the response.

**Figure 5:**
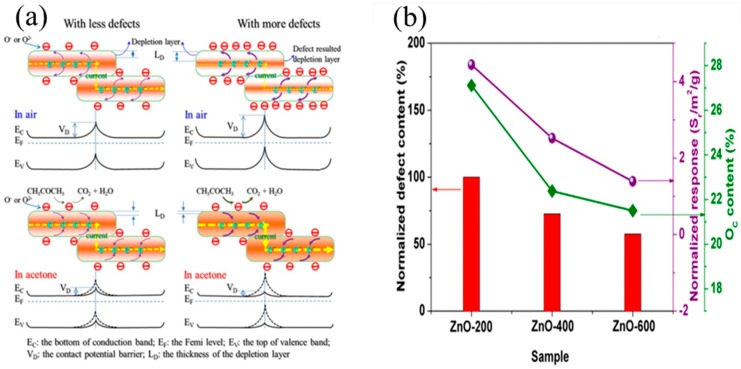
(**a**) Schematic illustration of the contact potential barrier under the electron transfer between two neighboring ZnO nanosheets viewed from the side with less and more defects; (**b**) The normalized defect content, the oxygen species content and the normalized response to 200 ppm acetone vapor at 300 °C. Reprinted from [78] with permission.

Subsequently, Li et al. studied the correlation between the sensor response and the surface defect content. The sensor response was based on the resistance change caused by the redox reaction between the target gases with the adsorbed oxygen species. The amount of adsorbed oxygen species played an important role in the enhancement of the sensor response. The surface defects could increase the amount of adsorbed oxygen species. ZnO nanosheets calcined at 200 °C exhibited abundant surface defects that could cause more oxygen molecules to be adsorbed on the metal oxide surfaces. This process also meant that more free electrons were captured from the conduction band, leading to a larger L_D_, as illustrated in the top right corner of Figure 5a. When the ZnO nanosheets were exposed to acetone gas, more acetone molecules reacted with the adsorbed oxygen species. More electrons were released to the conduction band, thus leading to a smaller L_D_ and inducing an improved performance, as presented in the bottom right corner of Figure 5a. It could be concluded that the sensor response was related to the surface defect content. As shown in Figure 5b, the normalized defect content decreased with increasing calcination temperature. This phenomenon could be explained by the fact that heat treatment reduced the number of defects. It is reasonable that the reduced number of defects could lead to diminished adsorbed oxygen species, leading to a decrease in the sensor response. Hence, the adsorbed oxygen species reached a maximum with greater surface defect content at the calcination temperature of 200 °C, and the sensor response reached a maximum as well.

### 4.3. Effect of the Catalyst

**Figure 6:**
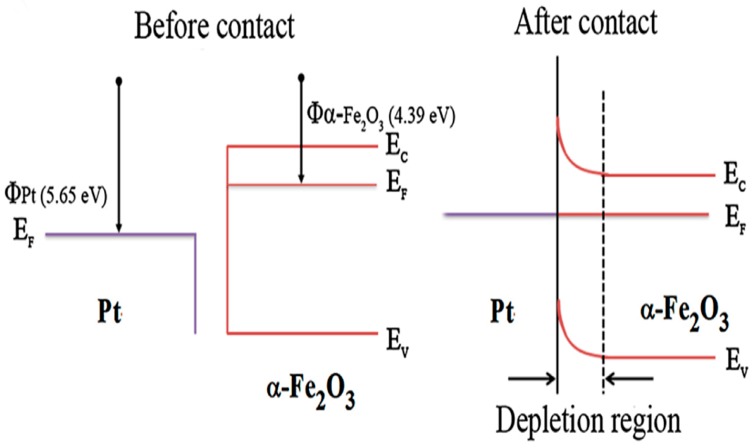
Schematic energy band diagrams of Pt and α-Fe_2_O_3_ before/after contact. Reprinted from [80] with permission.

The catalytic influence on semiconductor metal oxides results in the acceleration of the interaction between adsorbed oxygen species and target gases. Generally, noble metal is introduced into semiconductor metal oxides as a sensitizer or activator [79]. This is because the noble metal can affect the inter-granular contact region and thereby change the resistance of the gas sensor in two ways, namely, chemical sensitization and electronic sensitization. Liu et al. demonstrated that the sensor based on Pt-loaded α-Fe_2_O_3_ porous nanospheres exhibited a higher response to acetone than the sensor based on pure α-Fe_2_O_3_ samples [80]. For chemical sensitization, Pt was used as a catalyst to promote the formation of more active sites and the dissociation of oxygen molecules. The oxygen ions can be easily enriched and then spill over onto the surface of α-Fe_2_O_3_, extracting a greater degree of electrons. For electronic sensitization, Pt played the role of the electron acceptor on the surface of α-Fe_2_O_3_. The work function of Pt was higher than that of α-Fe_2_O_3_, and the electrons were transferred from α-Fe_2_O_3_ to Pt until equilibrium was reached, as shown in Figure 6. The process contributed to developing a wider electron-depleted region and greater band bending at the interface of Pt/α-Fe_2_O_3_. The incorporation of Pt into α-Fe_2_O_3_ increased the variation in resistance, resulting in an increase in the sensitivity. This finding verifies that noble metal can enhance the gas-sensing performance.

### 4.4. Effect of the Heterojunction

**Figure 7:**
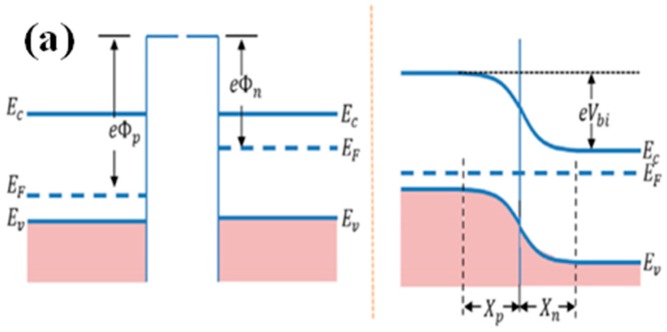

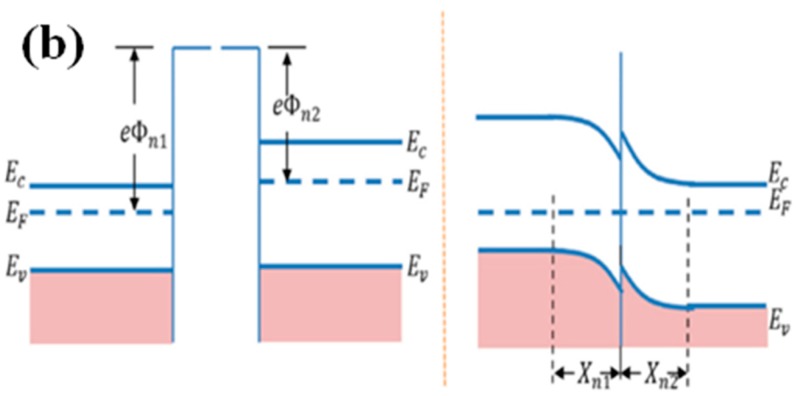
Schematic diagram showing the possible band structures at (**a**) p–n junction; (**b**) n–n junction. Reprinted from [25] with permission.

Heterostructures have attracted great interest due to the different combinations of p-type and n-type semiconductor metal oxides. The interface between two dissimilar semiconductor metal oxides is generally called a heterojunction. Commonly, there is a difference in the Fermi level between two dissimilar semiconductor metal oxides. The electrons flow from the high Fermi level to the low Fermi level until reaching equilibrium, as shown in Figure 7 [25]. This phenomenon can cause the formation of a depletion (or accumulation) region at the interface of heterostructures. The heterojunction can improve the sensitivity of sensors in two different types, including the p–n or n–p junction and the n–n or p–p junction.

#### 4.4.1. p–n or n–p Junction

Semiconductor metal oxides can be divided into two types, namely, p-type and n-type semiconductor metal oxides. There are two combinations of p-type and n-type semiconductor metal oxides in heterojunctions. One is the p–n junction in which an n-type semiconductor metal oxide aids p-type backbones. The other combination is an n–p junction in which a p-type semiconductor metal oxide aids n-type backbones. The major carriers of p-type semiconductors and n-type semiconductors are different. When two different types of semiconductors interact with each other, the electrons in the n-type semiconductors and holes in the p-type semiconductors diffuse in opposite directions [25]. This phenomenon induces a built-in voltage that impedes the flow of charge carriers, thereby increasing the resistance. Xiong et al. prepared ZnO/Co_3_O_4_ heterostructures with a highly enhanced ethanol sensing performance, which could be interpreted with the p-n heterojunction theory [81]. Due to the adsorption of oxygen, the hole density on the surface of Co_3_O_4_ increased, whereas the electron density on the surface of ZnO decreased. Hence, it became difficult for the holes to be transferred from Co_3_O_4_ to ZnO. Upon exposure to ethanol, electrons were released back to the conduction band, resulting in an increased electron density on the surface of ZnO and a decreased hole density on the surface of Co_3_O_4_. Thus, the number of holes transferred from Co_3_O_4_ to ZnO was increased, inducing a larger variation in resistance and triggering an enhanced ethanol sensing performance. In addition, the synergistic effect should also be taken into consideration due to the combination of the advantages of two different types of semiconductors. As a result, heterojunctions can improve the gas-sensing performance.

#### 4.4.2. n–n or p–p Junction

Heterojunction can also be formed by the same type of semiconductor metal oxide, namely, n-n or p–p junction. For the p–n junction, there are fewer free electrons at the interface due to electron-hole recombinations, leading to an increase in resistance. However, for the n–n junction, electrons are transferred from a high Fermi level to a low Fermi level, leading to the formation of an accumulation layer. The accumulation layer can be depleted by oxygen molecules, which further increases the potential energy barrier and sensitivity. For instance, Zhang et al. investigated the sensing performance of *α*-Fe_2_O_3_/SnO_2_ and SnO_2_ sensors to ethanol [82]. Notably, the *α*-Fe_2_O_3_/SnO_2_ sensors exhibited a higher response compared to SnO_2_ sensors due to the formation of heterojunctions. Heterostructures endowed them with an additional electron depletion layer at the interface of the heterojunctions, and changed the electron-transport properties.

### 4.5. Effect of the Humidity

**Figure 8:**
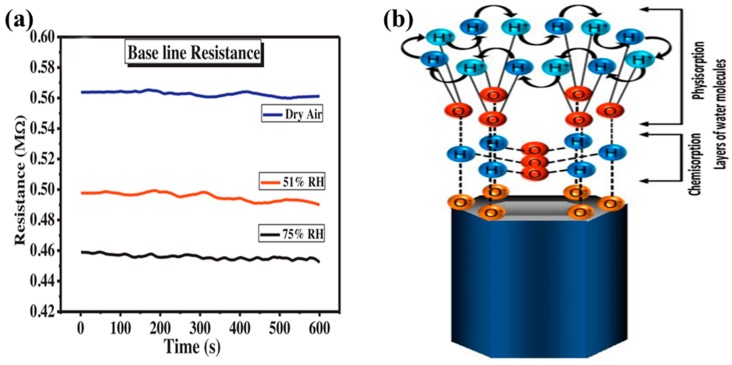
(**a**) Effect of relative humidity on the base line resistance; (**b**) Schematic representation of water molecule adsorption on ZnO nanotube surface. Reprinted from [83] with permission.

The environmental humidity plays an important role in affecting sensitivity. In fact, the humidity poses an enormous challenge for the development of semiconductor metal oxide gas sensors. This phenomenon can be explained by the large difference in performance between a dry and wet atmosphere. As shown in Figure 8a, the baseline resistance decreases with increasing humidity. It seems that the level of humidity determines the adsorption rate and thereby changes the sensitivity. The water molecules adsorbed on the semiconductor metal oxide surfaces are involved in the dissociative adsorption reaction and form hydroxyl (OH^−^) and hydrogen (H^+^) ions, as given in Equation (6):

(6) $$H_{2}O↔{\mathrm{OH}}^{-}+H^{+}$$

Acharyya et al. explained the conduction mechanism based on ZnO nanotubes under the influence of a humid environment, which was related to the chemisorption and physisorption of water molecules on the semiconductor metal oxide surfaces [83]. At a low humidity level, water molecules were chemisorbed at the active sites of semiconductor metal oxide surfaces, leading to the formation of hydroxyl groups absorbed on Zn cations and mobile protons. Moreover, protons reacted with adjacent surface O^2−^, resulting in secondary hydroxyl ions. The process decreased the baseline resistance in a low humidity environment compared to the case with dry air conditions. At a high humidity level, all active sites were occupied by chemisorbed water molecules, whereby the water molecules were physically adsorbed on the chemisorbed layer, as shown in Figure 8b. With increasing humidity, more multilayer physical adsorptions would be formed on the surface of the ZnO nanotube. Meanwhile, the water molecules in these layers could be ionized to form a large amount of hydronium (H_3_O^+^) ions under the electrostatic field. A charge transfer was induced by proton hopping between neighbouring water molecules. The process of proton conduction resulted in a lower baseline resistance compared to a low humidity level. Furthermore, water molecules competed with gas molecules at higher humidity level, which decreased the amount of charged oxygen species and hindered the adsorption probability of gas molecules. That is the reason why the sensitivity of gas sensors is reduced under humid conditions. Therefore, controlling the environmental humidity is important for improvement of the gas-sensing performance.

## 5. Semiconductor Metal Oxide Gas Sensors for the Detection of Volatile Organic Compounds

Volatile organic compounds (VOCs) originating from forest fires, vegetation emissions, agricultural respiration, painting, oil refining, vehicle exhaust emissions, and waste material disposal do increasingly serious harm to human life and health. In recent years, many efforts have been devoted to the development of high performance gas sensors for monitoring the air quality. Table 2, Table 3, Table 4, Table 5 and Table 6 list the gas-sensing performances of semiconductor metal oxides, which were influenced by the microstructure, defects, catalyst, heterojunction and humidity, towards ethanol, acetone, formaldehyde, toluene and acetylene. The details are discussed in the following five sections.

### 5.1. Ethanol Sensors

Ethanol has been widely used in the food industry, automotive fuel industry and manufacture of medicine, specifically for alcohol content monitoring and breath analysis. Drunk driving is extremely dangerous, so the detection of ethanol has an important significance of societal security [84]. Many researchers have investigated the detection of ethanol based on semiconductor metal oxide gas sensors. Table 2 lists the gas-sensing performances of semiconductor metal oxides for ethanol gas. Among the semiconductor metal oxides, SnO_2_-based nanomaterials have been considered promising candidates for detecting ethanol gas. Moreover, ternary oxides have also attracted extensive research interest for ethanol sensing.

**Figure 9:**
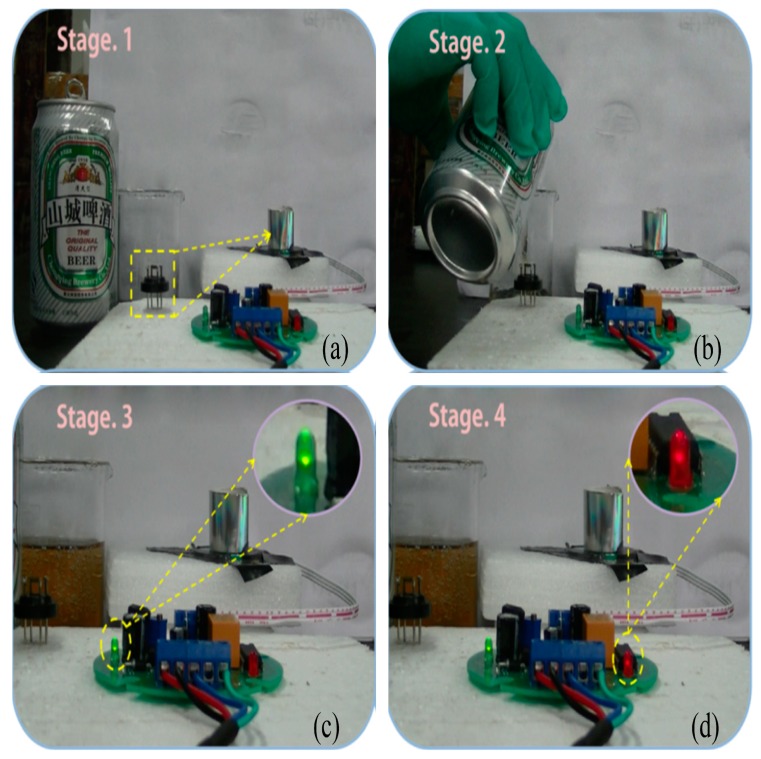
Demonstration of the beer monitoring using the mesoporous semi-blooming SnO_2_ nanoflowers-based sensor: (**a**) the beer was not poured; (**b**) the beer was poured; (**c**) the green diode turned on; (**d**) the red diode turned on. Reprinted from [102] with permission.

Li et al. prepared semi-blooming nanoflowers, blooming SnO_2_ nanoflowers and mesoporous semi-blooming SnO_2_ nanoflowers using hydrothermal conditions [102]. Among the three nanoflowers, mesoporous semi-blooming SnO_2_ nanoflowers were more advantageous to achieve a high response to 200 ppm ethanol due to the largest specific surface area and mesoporous structure. Their limit of detection reached 4.52 ppm. Therefore, the mesoporous semi-blooming SnO_2_ nanoflower-based sensor was applied to monitor the presence of beer, as shown in Figure 9. Figure 9a showed that two light-emitting diodes were off when beer was not poured. Then, a green diode would turn on when beer was poured. At the same time, a red diode also turned on, indicating the twinkling warning of the presence of beer, as shown in Figure 9b–d. The experimental results showed that fabricated semi-blooming nanoflower-based sensors can be considered attractive candidates for the determination of drunk driving behaviour.

Kim et al. reported Pt-doped SnO_2_ hollow nanospheres as an ethanol sensor [119]. The Pt-doped SnO_2_ hollow nanospheres were formed by Kirkendall diffusion. The response of the 0.3 wt.% Pt-doped SnO_2_ hollow nanosphere-based sensor was 1399.9 to 5 ppm ethanol, which showed a superior selectivity to ethanol due to the excellent catalytic ability of Pt. 

SnO_2_ nanowires coated with Fe_2_O_3_ nanoparticles were prepared by Choi et al. using the vapor-liquid-solid (VLS) process, a subsequent hydrothermal method and spin coating [95]. The reported results indicated that the operating temperature was an important factor for enhancing the gas-sensing performance. This finding was because the ionosorption of oxygen and ethanol gas was insufficient at low temperatures. However, the increment of carriers decreased the depletion layer of nanowires, leading to a decreased resistance at high temperatures. The Fe_2_O_3_ nanoparticle-coated SnO_2_ nanowires exhibited a high sensitivity to 200 ppm ethanol at 300 °C with negligible cross-responses to acetone, methanol, toluene and benzene compared to SnO_2_ nanowires. The improvement of the SnO_2_/Fe_2_O_3_ nanocomposite gas-sensing performance was caused by extending the width of the surface depletion layer at the interface of the heterojunctions.

Recently, ternary oxide-based ethanol sensors have been investigated by many researchers [85,109,118,123,124,125,126]. Zhou et al. prepared ZnSnO_3_ hollow cubes using the co-precipitation method, followed by alkali etching and annealing under inert conditions [109]. The sensor based on ZnSnO_3_ hollow cubes exhibited a superior response to 100 ppm ethanol at 260 °C, which was 1.77-fold higher than that of the ZnSnO_3_ solid cube-based sensor. The improved response value was ascribed to the hollow interior structure and porous surface, providing large surface area and excellent permeation. Furthermore, the ZnSnO_3_ hollow cube-based sensor exhibited a short response time of 4 s. However, the sensor had a relatively long recovery time of 276 s, attributed to the slow dissociation of oxygen and the formation of oxygen species (O^−^). Additionally, the ZnSnO_3_ hollow cube-based sensor showed remarkable selectivity to ethanol compared to other gases, such as C_6_H_6_, C_7_H_8_, NH_3_, CO and C_3_H_6_O.

Another ternary oxide has also been investigated for ethanol sensing. Choi et al. applied the polymerized complex method to prepare single-phase polycrystalline CuBi_2_O_4_ and investigated its gas-sensing property with the variation in the calcination temperature [85]. The CuBi_2_O_4_-based sensor with 500 °C-calcined powder was found to be sensitive to C_2_H_5_OH and showed the highest sensor response with the highest defect concentration. This was because the calcination temperature could change the intrinsic defect concentration, leading to changes in the hole concentration and electronic band gap energy with polaronic hopping conduction.

### 5.2. Acetone Sensors

Human breath is utilized as a bio marker for various kinds of diseases [127]. Acetone is the endogenous molecule that exists in human breath. The variation in concentration is an indication of disease. For patients with diabetes, acetone can be detected in their breath [128]. Owing to a lack of insulin within the body of the patient with diabetes, fat is used as energy rather than glucose. The accumulation of ketones during metabolic activity leads to a higher acetone concentration in the expiration gas of patients with diabetes. For a fast and easy diagnosis of diabetes, there have been many researchers who have focused on the detection of the least concentration of acetone in breath. Table 3 lists the acetone sensing performances of different semiconductor metal oxides. From most reported data, ZnO, ZnFe_2_O_4_ and Fe_2_O_3_ have been demonstrated to be the most promising potential materials for acetone sensing.

3D interconnected macro-mesoporous ZnO (3D-IMM-ZnO) nanostructures were prepared by the layer-by-layer filtration deposition method, which was exploited to detect acetone [156]. Liu et al. found that the sensor response value of 3D-IMM-ZnO nanostructures with the largest macropores was approximately 137, which was higher compared to ZnO nanoparticles and other 3D-IMM-ZnO nanostructures. The superior performance might be attributed to the macropores in the 3D-IMM-ZnO nanostructure accelerating gas accessibility, resulting in the acetone molecules sufficiently contacting the ZnO nanoparticles. In addition, 3D-IMM-ZnO nanostructures with the largest macropores could provide the largest cavities for gas diffusion, inducing faster electron transmission compared to other 3D-IMM-ZnO nanostructures. 

Zhang et al. synthesized ZnFe_2_O_4_ nanoparticles by tuning the mole ratio of ZnO to FeCl_3_ under hydrothermal conditions [133]. The mixture of ZnO and ZnFe_2_O_4_ was obtained by changing the mole ratio from 6:1 to 1:1. Notably, the content of ZnFe_2_O_4_ increased with decreasing mole ratio. When the mole ratio of ZnO to FeCl_3_ was 1:2, well-dispersed ZnFe_2_O_4_ nanoparticles with no other impurity phases were obtained for acetone sensing investigation. Compared to the precursor ZnO nanoparticle-based sensor, the ZnFe_2_O_4_ nanoparticle-based sensor reduced the power consumption. In addition, the ZnFe_2_O_4_ nanoparticle-based sensor exhibited improved gas-sensing properties, which were ascribed to the smaller size and higher surface area. 

**Figure 10:**
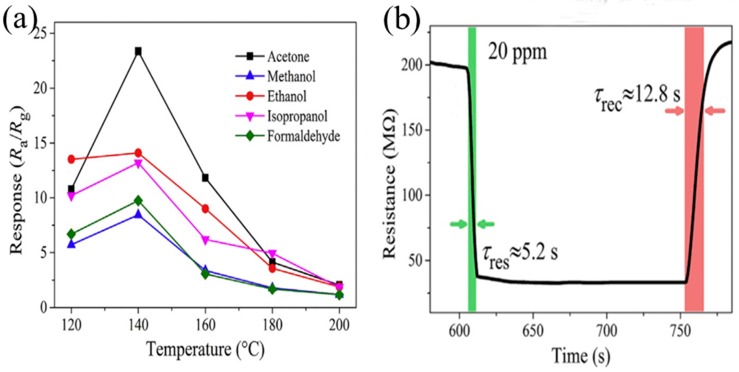
(**a**) Response of the sensor based on ZnO/ZnFe_2_O_4_ heterostructures to different gases (200 ppm) at different working temperatures; (**b**) Resistance transient of the sensor towards 20 ppm acetone. Reprinted from [154] with permission.

Song et al. reported triple-shell heterojunctional hollow microspheres for acetone detection based on a combination of ZnO and ZnFe_2_O_4_ [154]. The acetone sensing properties of ZnO/ZnFe_2_O_4_ heterostructures were investigated systematically. Apparently, the novel heterostructure exhibited the best response value towards acetone at an ultralow working temperature (140 °C) compared to other gases, as shown in Figure 10a. The enhanced response of ZnO/ZnFe_2_O_4_ triple-shell hollow microspheres was explained by the synergistic effect of ZnO and ZnFe_2_O_4_. The heterostructures ensured the effective electrical contact between ZnO and ZnFe_2_O_4_, which reduced the potential barrier and facilitated electron transport at low temperatures. This phenomenon prompted more adsorbed oxygen species to participate in the reaction, thereby improving the sensitivity. Figure 10b showed the resistance transient of the ZnO/ZnFe_2_O_4_ triple-shell hollow microsphere-based sensor towards 20 ppm acetone. The response and recovery times were approximately 5.2 and 12.8 s, respectively. The fast response/recovery speed was attributed to the porous and triple-shell hollow structures that were conducive to gas diffusion.

Additionally, the excellent sensing performance may be ascribed to three key factors proposed by Yamazoe et al., including the receptor function, the transducer function and the utility factor of the sensing body [161]. First, the multishell hollow structures of ZnO/ZnFe_2_O_4_ were receptive to more oxygen molecules, providing a significant reactive boost. Second, the spheres were composed of interconnected nanoparticles. The particle size was small enough and was twice the thickness of the space charge layer, thereby achieving complete depletion (transducer function). Third, the porous hollow structures and the voids between the three shells were favourable for the permeability and diffusion of acetone gases (utility factor), which was responsible for an improved response.

More recently, Fe_2_O_3_ has attracted much attention as a sensing material. Wang et al. prepared microsheet-assembled Fe_2_O_3_ microflowers by employing a hydrothermal method based on different concentrations of Fe^3+^ precursor solutions (0.025, 0.020, 0.015 and 0.010 mol/L Fe^3+^) [142]. The thickness of the microsheet became thinner with decreasing concentrations of Fe^3+^, increasing the porosity of the Fe_2_O_3_ microflowers. However, some of the microflowers could break up into particles and decreased in uniformity when the concentration of Fe^3+^ was 0.010 mol/L, which had agreat impact on the sensing performance. Thus, the 0.015 mol/L Fe^3+^ sample exhibited a high response with a value of 52 as well as a fast response and recovery times of 8 and 19 s, respectively. The excellent performance was attributed to the high surface area and numerous porous structures.

### 5.3. Formaldehyde Sensors

HCHO is considered one of the most common hazardous gases and is identified as a carcinogen. In daily life, many activities release varying amounts of HCHO into the atmosphere [162]. The release of formaldehyde not only harms health but also restricts sustainable development. To solve environmental problems, an increasing number of researchers have tried to develop high performance gas sensors for the detection of formaldehyde. In Table 4, we summarize the gas-sensing performances of various semiconductor metal oxides for detecting HCHO gas. Among them, In_2_O_3_, NiO and SnO_2_ are the most promising semiconductor metal oxides for the monitoring of formaldehyde.

Gu et al. investigated In_2_O_3_ nanoparticles for the improvement of formaldehyde sensing by manipulating the defect structure, such as the oxygen vacancies (V_O_) [165]. In_2_O_3_ nanoparticles with different contents of V_O_ were obtained by tuning the treatment time (10–30 min). In_2_O_3_-H10 exhibited the highest response (80) to formaldehyde with a fast response/recovery time (100 s/70 s). The excellent formaldehyde sensing performance was attributed to the greatest bulk quantity of V_O_. Moreover, In_2_O_3_-H10 had a narrower band gap and lower energy barrier due to the formation of new energy levels in the band gap induced by the increment in bulk V_O_ content, accelerating the electron mobility.

Zhang et al. reported an enhanced sensing performance of Au-loaded In_2_O_3_ porous nanocubes for formaldehyde detection [173]. The Au-loaded In_2_O_3_ porous nanocubes were fabricated by a hydrothermal method followed by thermal treatment. The sensitivity of Au-loaded In_2_O_3_ nanocubes was 2-fold higher than that of pure In_2_O_3_ nanocubes. Moreover, the incorporation of elemental Au reduced the optimum operating temperature. The improved sensing performance was explained by the effect of the catalyst including chemical sensitization and electronic sensitization. For the chemical sensitization, Au was added to In_2_O_3_ nanocubes as sensitizers. Owing to the spillover effect, it could promote more active sites on the surface of In_2_O_3_ nanocubes and induce more adsorption of oxygen molecules. For the electronic sensitization, Au acted as an electron acceptor on the surface of In_2_O_3_, leading to a broader electron-depleted region and greater band bending. Therefore, the incorporation of elemental Au was beneficial to an improved formaldehyde sensing performance.

Except for n-type semiconductor metal oxides, p-type semiconductor metal oxides for formaldehyde sensing have also received much attention owing to their suitable catalytic effect. For instance, Fu et al. prepared tetrahedron-like NiO nanostructures by a solvothermal reaction followed by calcination at different temperatures (400–600 °C) [175]. NiO nanotetrahedra annealed at 500 °C showed an excellent sensitivity to formaldehyde compared with other annealing temperatures. The sensitivity of p-type semiconductors was related to the concentration of cation vacancies. For the NiO nanotetrahedra, nickel vacancies were formed during the annealing of the NiO precursor. NiO nanotetrahedra annealed at 500 °C had a relatively higher nickel vacancy concentration, leading to improved sensitivity. Meanwhile, the sample had a relatively larger specific surface area, which was favourable for the adsorption of more oxygen molecules.

**Figure 11:**
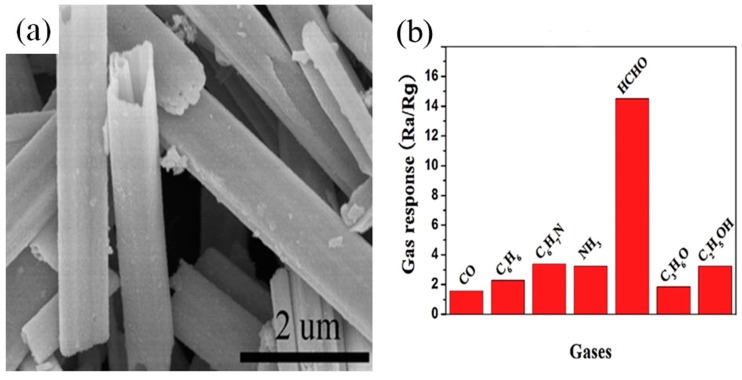
(**a**) SEM image of SnO_2_ microtubes; (**b**) Responses of the sensor to 50 ppm CO, C_6_H_6_, C_6_H_7_N, NH_3_, HCHO, C_3_H_6_O and C_2_H_5_OH operated at 92 °C. Reprinted from [168] with permission.

Most semiconductor metal oxides have superior responses to ppm-level formaldehyde at low working temperatures. However, the ppb-level concentration detection of HCHO at low working temperatures is more important for people due to threat it poses to the health. Recently, Zhang et al. applied a solvothermal method to synthesize SnO_2_ microtubes, which exhibited a low limit of detection (10 ppb) and a high response to HCHO at a low temperature of 92 °C with negligible cross-responses to CO, C_6_H_6_, C_6_H_7_N, NH_3_, C_3_H_6_O and C_2_H_5_OH, as shown in Figure 11 [168]. In addition, the sensor response of the microtubes was remarkably higher compared to irregular spheres, which was ascribed to the adsorption of more HCHO molecules caused by the hollow structure and rapid electron transfer provided by a direct path in the tubes.

### 5.4. Toluene Sensors

C_7_H_8_ is considered a kind of biomarker in human breath arising from the process of metabolism. Biomarkers can intuitively show abnormalities within the body. Lung cancer can be reflected by the gaseous toluene biomarker in exhaled breath [183]. Thus, toluene sensors can be used for non-invasive disease diagnostics through the detection of gaseous biomarkers. Recently, many gas sensors have been developed in the field of detecting toluene. As shown in Table 5, SnO_2_-based nanostructures have often been investigated for the detection of toluene. Great efforts surrounding the addition of noble metals and metal oxides to SnO_2_ nanomaterials have been devoted to improving the toluene sensing performance.

**Figure 12:**
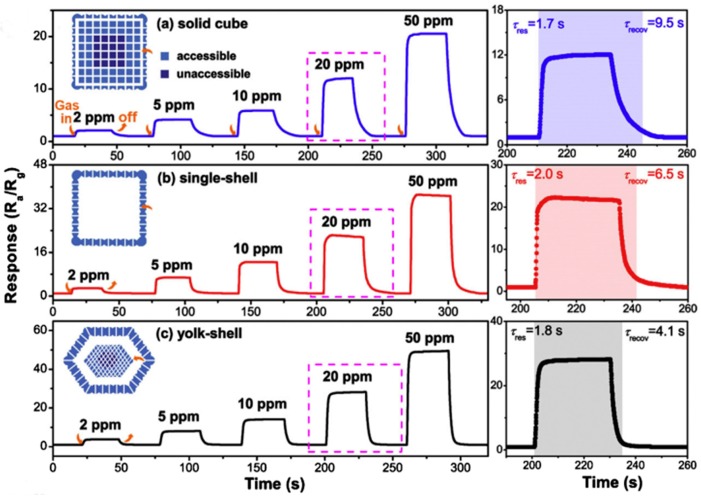
Dynamic toluene sensing transients of SnO_2_ products with (**a**) solid cubes; (**b**) single-shell structures; (**c**) yolk-shell structures to toluene with different concentrations. The right insets show the corresponding response time (res) and recovery time (recov) examined to 20 ppm toluene, respectively. Reprinted from [201] with permission.

Bing et al. reported the synthesis of solid SnO_2_ cubes, single-shell SnO_2_ cages and yolk-shell SnO_2_ cuboctahedras for the detection of toluene [201]. The sensor response towards 20 ppm toluene at 250 °C was increased from 12.1 for solid SnO_2_ cubes and 22.4 for single-shell SnO_2_ cages to 28.6 for yolk-shell SnO_2_ cuboctahedra, as shown in Figure 12. In addition, yolk-shell SnO_2_ cuboctahedra exhibited the fastest response and recovery times of 1.8 and 4.1 s, respectively. 

The exceptional gas-sensing performance of yolk-shell SnO_2_ cuboctahedra was attributed to a special hierarchical architecture with a core@void@shell configuration. The distinctive structures provided penetrable shells and cores for fast mass transfer as well as multiple walls for an increased surface area.

Xie et al. synthesized pristine SnO_2_, pristine Pd/SnO_2_ and carbonized Pd/SnO_2_ nanofibers by electrospinning for toluene sensing [185]. They reported that the pristine Pd/SnO_2_ sensors exhibited a higher response and lower operating temperature compared to the pristine SnO_2_ sensors due to the chemical and electronic sensitizations caused by the catalyst Pd. After carbonization treatment, the carbonized Pd/SnO_2_ nanofibers showed a higher response compared to the pristine Pd/SnO_2_ nanofibers owing to the porous structure and the enhancement of the chemisorbed oxygen (O_c_). On the one hand, Pd/SnO_2_ nanofibers with carbonization possessed porous structures that were beneficial for gas diffusion into the inner-surface/outer-surface. On the other hand, the oxidation and reduction reaction of carbon species might lower the oxygen supply to Sn during the formation of SnO_2_ particles. Moreover, the combustion of carbon species might also consume a portion of the oxygen atoms coming from the SnO_2_ particles. Hence, more chemisorbed oxygens could participate in the reaction due to the formation of more oxygen defects compared with the traditional method, thereby enhancing the sensor response to toluene.

Kim et al. reported the exceptional sensing performance of Pt-functionalized SnO_2_/ZnO core/shell nanowires for detecting ppb-levels of toluene [187]. To verify the superior toluene sensing characteristics of Pt-functionalized SnO_2_/ZnO core/shell nanowires, ZnO nanowires, SnO_2_ nanowires and SnO_2_/ZnO core/shell nanowires were also prepared for the detection of toluene. After comparison, it could be found that the SnO_2_/ZnO core/shell nanowires showed enhanced properties compared to the two single component semiconductor metal oxide nanowires due to the formation of a heterointerface between SnO_2_ and ZnO. The sensor response of the heterostructure varied with the thickness of the shell due to the radial modulation of the electron-depleted shell layer. Therefore, the toluene response was further improved by controlling the thickness of the shell. Furthermore, Pt-functionalized SnO_2_/ZnO core/shell nanowires with a shell thickness of 80 nm exhibited an excellent sensor response value of 279 for 100 ppb toluene and outstanding selectivity in the presence of interfering gases including CO, CO_2_, and C_6_H_6_. The catalytic effect of Pt nanoparticles might not only promote more interactions between the gaseous molecules but also dissociate the toluene gas more effectively relative to the other interfering gases. Moreover, the attachment of Pt nanoparticles to the ZnO shell layer could cause the flow of electrons from the ZnO shell layer to the Pt nanoparticles according to the Fermi level difference, leading to a further improvement of the sensing performance.

### 5.5. Acetylene Sensors

Acetylene is an explosive and flammable volatile organic compound. Therefore, it is of great significance to detect acetylene gas in industrial production, chemical processing, cutting metals and welding. For environmental monitoring and safety protection, the development of high efficiency acetylene sensors has drawn attention [203,204]. The acetylene sensing performances of semiconductor metal oxides are rarely reported but highly meaningful. Table 6 summarizes the acetylene sensing properties of semiconductor metal oxides. ZnO is the most promising candidate for acetylene sensors.

Qiao et al. prepared mesoporous ZnO nanosheets by the hydrothermal method [205]. They reported that ZnO nanosheets-based sensors exhibited superior acetylene sensing performance at the optimum operating temperature of 400 °C with a response (R_a_/R_g_) of 164.3 for 200 ppm acetylene. In particular, the sensor could detect 1 ppm acetylene with a high response value (R_a_/R_g_ = 10). In addition, it was noteworthy that a level of 1 ppm acetylene could be detected at 285 °C below the autoignition temperature of acetylene (305 °C) with a response value of 4, which was high enough for practical applications (R_a_/R_g_ ≥ 3).

The structural sensitization involved morphology and defect effects, enhancing the gas-sensing performance of the ZnO nanosheet-based sensors. First, mesoporous nanosheets possessed large specific surface area and mesoporous feature, contributing to the increased adsorption capacity of gas molecules and improved gas diffusion. Additionally, the thickness of the ZnO nanosheets was nearly identical to two times the Debye length, leading to almost fully depleted electrons and a remarkably enhanced sensor response. Second, the abundant intrinsic defects present in the mesoporous ZnO nanosheets could absorb more oxygen molecules and subsequently ionize them. It meant that a broader depletion layer was generated, resulting in an enhanced acetylene sensing property.

**Figure 13:**
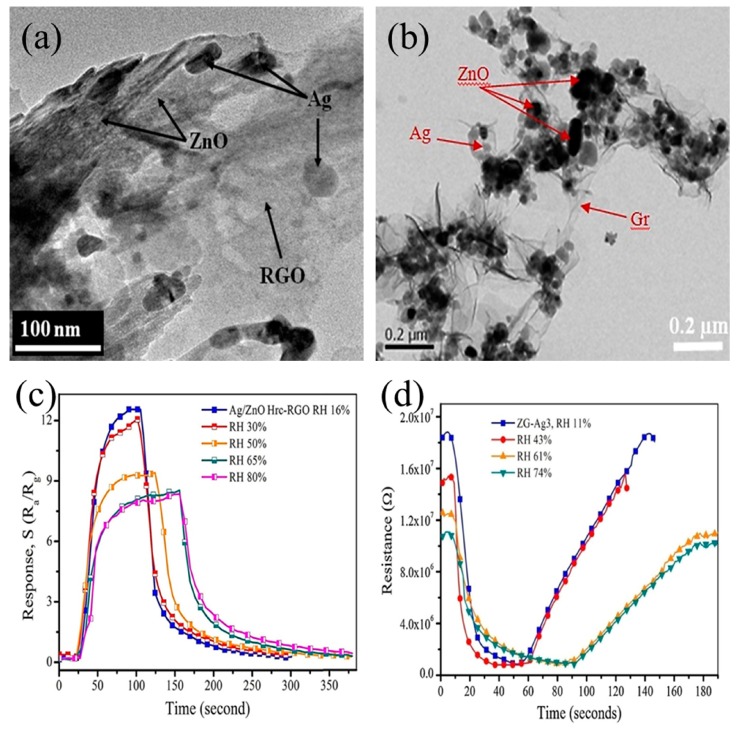
TEM images of (**a**) Ag-loaded hierarchical ZnO-reduced graphene oxide hybrid; (**b**) Ag-loaded ZnO-reduced graphene oxide hybrid; Transient response at different humidity concentrations of (**c**) Ag-loaded hierarchical ZnO-reduced graphene oxide hybrid; (**d**) Ag-loaded ZnO-reduced graphene oxide hybrid. Reprinted from [207,215] with permission.

Uddin et al. investigated Ag-loaded hierarchical ZnO-reduced graphene oxide (Ag/ZnO Hrc-RGO) hybrids and Ag-loaded ZnO-reduced graphene oxide (Ag/ZnO-Gr) hybrids as sensing materials for acetylene sensing at low temperatures, as shown in Figure 13a,b [207,215]. They found that the Ag/ZnO-Gr hybrid nanostructures exhibited a higher response value and faster response time compared to the Ag/ZnO Hrc-RGO hybrid nanostructures. The higher response could be explained by the ZnO nanoparticles in the Ag/ZnO-Gr hybrids having larger surface area, more uniform shape, smaller grain size and better crystallinity compared with the ZnO hierarchical nanostructures in the Ag/ZnO Hrc-RGO hybrids. In addition, the enhanced sensing property of the Ag/ZnO-Gr hybrids towards acetylene might be caused by the improved contacts among the Ag nanoparticles, ZnO nanoparticles and thin nanosheets of the reduced graphene oxides.

In addition, they also reported the humidity effect on the gas-sensing properties of Ag/ZnO-Gr hybrid nanostructures and Ag/ZnO Hrc-RGO hybrid nanostructures, as shown in Figure 13c,d. It should be noted that water molecules could affect the properties of gas sensors. The response decreased with increasing RH concentration. This phenomenon was because water molecules decreased the adsorption of chemisorbed oxygen species, resulting in chemisorbed hydroxide (OH^−^) on the surface of sensing materials. The process reduced the surface area and decreased the C_2_H_2_ adsorption, leading to deterioration of the response.

## 6. Conclusions and Outlook

In this review, the general properties of VOCs and the gas-sensing mechanism of chemoresistive semiconductor metal oxide gas sensors are described to better understand the necessity of detecting VOCs and the operating principle of gas sensors. Then, five factors that affect the sensitivity, including the microstructure, defects, catalyst, heterojunction and humidity, were introduced. Subsequently, the developments of semiconductor metal oxide gas sensors for detecting five common VOCs, such as ethanol, acetone, formaldehyde, toluene and acetylene, were reviewed from the perspective of the five factors above. From most reported data, it can be concluded that ZnO, SnO_2_, Fe_2_O_3_, In_2_O_3_ and NiO are the most promising semiconductor metal oxides for detecting VOCs.

Although many researchers have committed to fabricating high performance gas sensors, there is still room for further improvement of the gas sensitivity. Except for the sensing materials, gas sensor devices need to be improved for practical applications, particularly with respect to reliability and durability. Accuracy and robustness tests should also be carried out for the sake of security. With the global environment worsening, it is imperative that continuous research should be conducted to improve the sensor performance in detecting hazardous gases. Undoubtedly, the improvement of gas sensors will continue to play an important role in the future.

## Figures and Tables

**Table 1:** Chemical formula, formula weight, IDLH (immediately dangerous to life or health) and TLV (threshold limit value) of VOCs.

Gases	Chemical Formula	Formula Weight (g/mol)	IDLH (ppm)	TLV (ppm)
Ethanol	C_2_H_5_OH	46.07	3300	1000
Acetone	CH_3_COCH_3_	58.08	20,000	750
Formaldehyde	HCHO	30.03	20	0.75
Toluene	C_7_H_8_	92.14	500	100
Acetylene	C_2_H_2_	26.04	NA	NA

NA = not available.

**Table 2:** Summary of the gas-sensing performances of semiconductor metal oxides for ethanol gas.

Order	Dimensions	Materials	Synthesis Method	Conc. (ppm)	LOD (ppm)	Temp. (°C)	τ_res_ (s)	τ_rec_ (s)	Resp.	Ref.
1	0D	CuBi_2_O_4_ powders	polymerized complex method	1000	5	400	57	294	10.4 ^b^	[85]
2		SnO_2_ nanoparticles	microwave treatment	250	NA	100	16	25	30 ^c^	[86]
3	1D	Co_3_O_4_ microrods	interfacial-reaction	100	NA	220	0.8	10.8	9.8 ^b^	[87]
4		CuO/In_2_O_3_ nanorods	thermal evaporation and sputtering	50	NA	300	53	149	3.82 ^a^	[88]
5		In_2_O_3_ microrods	hydrothermal	100	1	300	15	20	18.33 ^a^	[89]
6		LaFeO_3_ nanotubes	electrospinning	100	NA	160	2	4	9.4 ^b^	[90]
7		Pd/Fe_2_O_3_ nanotubes	electrospinning	50	0.1	240	8	30	65.4 ^a^	[91]
8		ZnO nanotubes	electrodeposition and electrochemical etching	700	1	RT	4.56 min	1.53 min	64.17% ^c^	[83]
9		LaMnO_3_/SnO_2_ nanofibers	electrospinning	100	NA	260	6	34	20 ^a^	[92]
10		WO_3_/SnO_2_ nanofibers	coaxial electrospinning	10	NA	280	18.5	282	5.09 ^a^	[93]
11		ZnO nanowires	solvothermal	500	NA	340	6	26	10.68 ^a^	[94]
12		SnO_2_/Fe_2_O_3_ nanowires	VLS, hydrothermal and spin coating	5	NA	300	100	300	3.07 ^a^	[95]
13		SnO_2_/ZnO nanowires	carbon-assisted thermal evaporation	400	NA	400	NA	NA	128 ^c^	[96]
14		SnO_2_/ZnO nanowires	thermal evaporation and spray-coating	100	NA	400	NA	NA	14.1^a^	[97]
15	2D	CuO films	RF sputtering	12.5	NA	180	31	52	2.2 ^b^	[98]
16		Pd/Ce/SnO_2_ films	co-precipitation	100	NA	250	6	20	88 ^c^	[99]
17		NiO nanosheets	hydrothermal	50	1	240	4	7	11.15 ^b^	[100]
18		ZnO nanosheets	hydrothermal	50	1	330	15	12	83.6 ^a^	[101]
19	3D	SnO_2_ nanoflowers	hydrothermal	200	4.52	240	10	16	62.2 ^a^	[102]
20		SnO_2_ nanoflowers	hydrothermal	100	NA	300	10	16	47.29 ^a^	[103]
21		Au/SnO_2_ hierarchical structures	hydrothermal	100	NA	340	5	10	18 ^a^	[104]
22		NiO hierarchical structures	hydrothermal	400	NA	300	4	8	32 ^b^	[105]
23		SnO_2_ macropores structures	sol-gel method	500	NA	240	NA	NA	70.94 ^a^	[106]
24		MoO_3_ microboxes	hydrothermal	100	1	260	15	5	78 ^a^	[107]
25		SnO_2_ nanocubes	hydrothermal	100	1	200	23	21	1670.5 ^a^	[108]
26		ZnSnO_3_ nanocubes	co-precipitation	100	NA	260	4	276	34.1 ^a^	[109]
27		PdO/Zn_2_SnO_4_ octahedrons	hydrothermal and wet impregnation treatment	100	0.5	250	1	206	82.3 ^a^	[110]
28		WO_3_ urchin-like structures	hydrothermal	100	NA	350	28	12	68.56 ^a^	[111]
29		Co_3_O_4_ microspheres	interfacial-reaction	100	1	220	0.1	0.7	38.2 ^b^	[112]
30		CuO microspheres	precipitation	400	NA	250	17	11.9	5.6 ^b^	[113]
31		CuO microspheres	hydrothermal	100	NA	200	2	8	390% c	[114]
32		Fe_2_O_3_/Co_3_O_4_ microspheres	solution route	100	NA	170	3.3	5.4	16.1 ^b^	[115]
33		SnO_2_ microspheres	ion exchange method	200	NA	260	NA	NA	103.1 ^a^	[116]
34		SnO_2_/Fe_2_O_3_ microspheres	hydrothermal	100	0.1	260	3	4	41.7 ^a^	[117]
35		ZnFe_2_O_4_ microspheres	solvothermal	10	0.5	180	5.1	6.5	6.85 ^a^	[118]
36		Pt/SnO_2_ nanospheres	spray drying and Kirkendall diffusion	5	0.25	325	1	1577	1399.9 ^a^	[119]
37		SnO_2_ nanospheres	precipitation	200	NA	260	10	8	274.5 ^a^	[120]
38		SnO_2_ nanospheres	hydrothermal	100	0.5	350	5	NA	10.5 ^a^	[121]
39		SnO_2_/ZnO nanospheres	self-sacrificial template	50	NA	270	0.4	235	7.5 ^a^	[122]
40		ZnSnO_3_ nanospheres	hydrothermal	100	NA	200	4	30	32 ^a^	[123]
41		Zn_2_SnO_4_ nanospheres	hydrothermal	50	5	180	NA	NA	23.4 ^a^	[124]

Note: Conc. = concentration; LOD = limit of detection; Temp. = temperature; τ_res_ = response time; τ_cov_ = recovery time; Resp. = Response; Ref. = References; RT = room temperature; ^a^ S = R_a_/R_g_; ^b^ S = R_g_/R_a_; ^c^ S = (ΔR/R_a_)*100%; ^d^ S = I_g_/I_a_; NA = not available.

**Table 3:** Summary of the gas-sensing performances of semiconductor metal oxides for acetone gas.

Order	Dimensions	Materials	Synthesis Method	Conc. (ppm)	LOD (ppm)	Temp. (°C)	τ_res_ (s)	τ_rec_ (s)	Resp.	Ref.
1	0D	Au/ZnO nanoparticles	MOF template	10	0.05	280	15	12	43 ^a^	[129]
2		Ce/CoFe_2_O_4_ nanocrystallites	molten-salt	2000	NA	200	38	61	157 ^b^	[130]
3		Fe_2_O_3_ nanoparticles	hard template	100	NA	300	6	NA	26.3 ^a^	[131]
4		Pt/In_2_O_3_ nanoparticles	sol-gel	1.56	0.01	200	25	120	12 ^a^	[132]
5		ZnFe_2_O_4_ nanoparticles	hydrothermal	200	NA	200	NA	NA	39.5 ^a^	[133]
6		LaFeO_3_ powders	sol-gel method	10	NA	200	21	6	7.83 ^b^	[134]
7	1D	Co_3_O_4_ nanochains	solution route	200	5	180	32	35	10.5 ^b^	[135]
8		Fe_2_O_3_ nanorods	interfacial-reaction	100	NA	280	0.4	2.4	32.5 ^a^	[136]
9		ZnFe_2_O_4_ nanorods	hydrothermal	100	NA	260	1	11	52.8 ^a^	[137]
10		Fe_2_O_3_ nanotubes	electrospinning	100	NA	240	9	3	11 ^a^	[138]
11	2D	Co_3_O_4_ nanosheets	fluorine-assisted hydrothermal	1000	NA	111	NA	NA	36.5 ^b^	[139]
12		ZnO nanosheets	precipitation	200	0.81	300	18.71	13.75	106.1 ^a^	[78]
13		In_2_O_3_ films	sparking process	2000	NA	350	3	NA	117 ^a^	[140]
14		ZnO films	sol-gel dip coating	50	2	RT	60	28	490 ^a^	[141]
15		ZnO films	e-beam and post-annealing	100	10	280	6	18	30 ^a^	[9]
16	3D	Fe_2_O_3_ microflowers	hydrothermal	100	NA	220	8	19	52 ^a^	[142]
17		SnO_2_ nanoflowers	hydrothermal	50	NA	170	3	28	29.2 ^a^	[143]
18		ZnO nanoflowers	hydrothermal	100	NA	300	7	NA	18.6 ^a^	[144]
19		ZnFe_2_O_4_/ZnO microflowers	solution route	50	1	250	2	25	8.3 ^a^	[145]
20		ZnO porous flowers	hydrothermal	50	0.25	280	2	23	97.8 ^a^	[146]
21		LaFeO_3_ microspheres	hydrothermal	100	1	225	5	25	25.5 ^b^	[147]
22		Au/In_2_O_3_ nanospheres	hydrothermal	10	NA	320	4	9	53.08 ^b^	[148]
23		LaFeO_3_ microspheres	hydrothermal	100	NA	260	9	17	29 ^b^	[149]
24		SnO_2_/Fe_2_O_3_ nanospheres	self-sacrificial template	100	NA	260	3	6	47.1 ^a^	[150]
25		ZnFe_2_O_4_ nanospheres	solvothermal	30	0.8	200	9	272	11.8 ^a^	[151]
26		ZnFe_2_O_4_ microspheres	solvothermal	50	0. 5	200	NA	NA	28.3 ^a^	[152]
27		ZnFe_2_O_4_ microspheres	hydrothermal	20	0.13	206	NA	NA	13.6 ^a^	[153]
28		ZnO/ZnFe_2_O_4_ microspheres	self-sacrificial template	20	NA	140	5.2	12.8	5.9 ^a^	[154]
29		SnO_2_ hierarchical structures	hydrothermal	100	0.05	325	NA	NA	175 ^a^	[155]
30		ZnO macro-mesoporous structures	layer-by-layer filtration deposition	100	NA	260	13	50	137 ^a^	[156]
31		ZnO urchin-like structures	hydrothermal	10	NA	300	42	10	58.1 ^a^	[157]
32		ZnO/ZnFe_2_O_4_ microcubes	MOF template	5	0.5	250	5.6 min	6.0 min	9.4 ^a^	[158]
33		ZnFe_2_O_4_ octahedral nanocages	thermal decomposition	200	NA	120	NA	NA	64.4 ^a^	[159]
34		ZnO/ZnFe_2_O_4_ nanocages	self-sacrificial template	100	1	290	8	32	25.8 ^a^	[160]

Note: Conc. = concentration; LOD = limit of detection; Temp. = temperature; τ_res_ = response time; τ_cov_ = recovery time; Resp. = Response; Ref. = References; RT = room temperature; ^a^ S = R_a_/R_g_; ^b^ S = R_g_/R_a_; ^c^ S = (ΔR/R_a_)*100%; ^d^ S = I_g_/I_a_; NA = not available.

**Table 4:** Summary of the gas-sensing performances of semiconductor metal oxides for formaldehyde gas.

Order	Dimensions	Materials	Synthesis Method	Conc. (ppm)	LOD (ppm)	Temp. (°C)	τ_res_ (s)	τ_rec_ (s)	Resp.	Ref.
1	0D	In_2_O_3_ particles	hydrothermal	50	NA	130	37	48	86 ^a^	[163]
2		In_2_O_3_ nanoparticles	hydrothermal	10	NA	280	4	8	20 ^a^	[164]
3		In_2_O_3_ nanoparticles	hydrothermal	100	NA	230	100	70	80 ^a^	[165]
4		NiO nanoparticles	electrodeposition	1	NA	230	53.7	13.3	3.43 ^b^	[166]
5	1D	NiO nanochains	electrospinning	50	1	210	1	10	NA	[167]
6		SnO_2_ microtubes	solvothermal	100	0.01	92	NA	NA	26.2 ^a^	[168]
7	2D	ZnO nanosheets	hydrothermal	50	NA	350	9	11	37.8 ^a^	[169]
8	3D	CeO_2_ polyhedron	hydrothermal	150	1	220	21	16	172 ^a^	[170]
9		In_2_O_3_ polyhedrons	solution route	20	NA	240	1	2	8.2 ^a^	[171]
10		SnO_2_ cedar-like structures	hydrothermal	100	1	200	1	13	13.3 ^a^	[172]
11		Au/In_2_O_3_ nanocubes	hydrothermal	100	NA	240	3	8	37 ^b^	[173]
12		ZnSnO_3_ multishelled cubes	coprecipitation and alkaline etching route	100	NA	220	1	59	37.2 ^a^	[174]
13		NiO nanotetrahedra	solvothermal	50	NA	250	NA	NA	11.6 ^b^	[175]
14		In_2_O_3_ hierarchical structures	solution route	100	1	260	1	8	8.6 ^a^	[176]
15		In_2_O_3_ microflowers	hydrothermal/solvothermal	50	0.5	190	25	65	90 ^a^	[177]
16		NiO microflowers	solvothermal	100	NA	200	30	56	3.5 ^b^	[178]
17		CuO microspheres	hydrothermal	100	NA	300	26	28	3.2 ^b^	[179]
18		In_2_O_3_ microspheres	thermal decomposition	50	10	175	25	104	30 ^a^	[180]
19		In_2_O_3_ microspheres	self-sacrificial template	100	NA	300	21	7	40.9 ^a^	[181]
20		SnO_2_ microspheres	hydrothermal	200	1	300	NA	NA	9 ^a^	[182]

Note: Conc. = concentration; LOD = limit of detection; Temp. = temperature; τ_res_ = response time; τ_cov_ = recovery time; Resp. = Response; Ref. = References; RT = room temperature; ^a^ S = R_a_/R_g_; ^b^ S = R_g_/R_a_; ^c^ S = (ΔR/R_a_)*100%; ^d^ S =I_g_/I_a_; NA = not available.

**Table 5:** Summary of the gas-sensing performances of semiconductor metal oxides for toluene gas.

Order	Dimensions	Materials	Synthesis Method	Conc. (ppm)	LOD (ppm)	Temp. (°C)	τ_res_ (s)	τ_rec_ (s)	Resp.	Ref.
1	0D	Au/ZnO nanoparticles	coprecipitation	100	NA	377	NA	6 min	92 ^a^	[184]
2	1D	Pd/SnO_2_ nanofibers	electrospinning and carbonization	100	0.5	250	3	35	24.6 ^a^	[185]
3		Pd/WO_3_ nanofibers	electrospinning	1	0.02	350	10.9	16.1	5.5 ^b^	[186]
4		Pt/SnO_2_/ZnO nanowires	vapor-liquid-solid, atomic layer deposition and γ-ray radiolysis	0.1	NA	300	NA	NA	279 ^a^	[187]
5		TiO_2_ nanotubes	hydrothermal	50	NA	500	110–130	800–1150	3 ^a^	[188]
6		ZnO nanowires	electrospinning and hydrothermal	100	1	240	9	4	12.7 ^a^	[189]
7		Fe_2_O_3_/NiO nanotubes	hydrothermal	5	0.5	275	10	24	8.8 ^b^	[190]
8		SnO_2_/Fe_2_O_3_ nanotubes	electrospinning	50	0.05	260	5	11	25.3 ^a^	[191]
9		Fe_2_O_3_/SnO_2_ nanowires	ultrasonic spray pyrolysis and hydrothermal	100	NA	90	20	15	49.7% ^c^	[192]
10		Co_3_O_4_ nanorods	solvothermal	200	NA	200	90	55	35 ^b^	[193]
11		MoO_3_/Fe_2_(MoO_4_)_3_ nanobelts	hydrothermal	50	NA	250	NA	NA	5.3 ^a^	[194]
12	3D	Pd/SnO_2_ microspheres	hydrothermal	20	0.1	230	0.48	5.5	52.9 ^a^	[195]
13		In_2_O_3_ microspheres	hydrothermal	50	0.5	350	12	25	85% ^c^	[196]
14		Au/TiO_2_ pecan-kernel-like structures	hydrothermal and precipitation	100	NA	375	4	5	7.3 ^a^	[197]
15		Pd/SnO_2_ cubic nanocages	self-sacrificial template and precipitation	20	0.1	230	0.4	16.5	41.4 ^a^	[198]
16		SnO_2_ nanocages	self-sacrificial template	20	NA	250	2.3	5.8	33.4 ^a^	[199]
17		PdO/ZnO nanoflowers	hydrothermal	100	NA	240	1	9	10.9 ^a^	[200]
18		SnO_2_/SnO_2_ cuboctahedra	anneal-etching	20	NA	250	1.8	4.1	28.6 ^a^	[201]
19		ZnFe_2_O_4_ nanospheres	nonaqueous route	100	1	300	18.14	29.2	9.98 ^a^	[202]

Note: Conc. = concentration; LOD = limit of detection; Temp. = temperature; τ_res_ = response time; τ_cov_ = recovery time; Resp. = Response; Ref. = References; RT = room temperature; ^a^ S = R_a_/R_g_; ^b^ S = R_g_/R_a_; ^c^ S = (ΔR/R_a_)*100%; ^d^ S = I_g_/I_a_; NA = not available.

**Table 6:** Summary of the gas-sensing performances of semiconductor metal oxides for acetylene gas.

Order	Dimensions	Materials	Synthesis Method	Conc. (ppm)	LOD (ppm)	Temp. (°C)	τ_res_ (s)	τ_rec_ (s)	Resp.	Ref.
1	0D	Pt/ZnO nanoparticles	flame spray pyrolysis	10000	NA	300	6	60	836 ^a^	[206]
2		Ag/ZnO/RGO hybrid nanostructures	chemical route	100	1	150	25	80	21.2 ^a^	[207]
3	1D	Sm_2_O_3_/SnO_2_ nanorods	hydrothermal	100	NA	260	8	9	50.5 ^a^	[208]
4		WO_3_ nanowires	hydrothermal	200	NA	300	6	7	58 ^a^	[209]
5		Au/ZnO/In_2_O_3_ belt-tooth	chemical vapor deposition	100	NA	90	8.5	NA	5 ^a^	[210]
6	2D	ZnO nanosheets	hydrothermal	100	1	400	11	5	101.1 ^a^	[205]
7		Au/ZnO nanorings	hydrothermal and deposition	100	0.5	255	4	3	28 ^a^	[211]
8	3D	ZnO nanoflowers	hydrothermal	200μL/L	NA	375	8	11	48.2 ^a^	[212]
9		Au/ZnO microspheres	hydrothermal and precipitation	100	1	183.5	8	2	311.3 ^a^	[213]
10		NiO/SnO_2_ hierarchical structures	hydrothermal	100	1	206	2	5	13.8 ^a^	[214]
11		Ag/ZnO/RGO hierarchical structures	photochemical	100	3	200	57	90	12.3 ^a^	[215]

Note: Conc. = concentration; LOD = limit of detection; Temp. = temperature; τ_res_ = response time; τ_cov_ = recovery time; Resp. = Response; Ref. = References; RT = room temperature; ^a^ S = R_a_/R_g_; ^b^ S = R_g_/R_a_; ^c^ S = (ΔR/R_a_)*100%; ^d^ S = I_g_/I_a_; NA = not available.
